# Preliminary study on *Cyclocodon lancifolius* leaf blight and screening of *Bacillus subtilis* as a biocontrol agent

**DOI:** 10.3389/fmicb.2024.1459868

**Published:** 2024-10-15

**Authors:** Xin Yang, You-chao Dang, Jing-zhong Chen, Ke-cheng Xu, Dao-die Dai, Qing-wen Sun

**Affiliations:** ^1^College of Pharmacy, Guizhou University of Traditional Chinese Medicine, Guiyang, China; ^2^Guizhou Key Laboratory for Raw Material of Traditional Chinese Medicine Guiyang, Guiyang, China; ^3^Resource and Development Research Center of C. lancifolius, Guizhou University of Traditional Chinese Medicine, Guiyang, China

**Keywords:** *Stemphylium lycopersici*, growth characteristics, synthetic fungicide, enzyme, bacterium

## Abstract

**Introduction:**

This study aimed to identify the pathogen responsible for leaf blight in *Cyclocodon lancifolius*, investigate its biological characteristics, and identify effective synthetic fungicides. Additionally, this study examined changes in physiological and biochemical indices of leaves following pathogen infection and screened biocontrol bacteria that inhibit the pathogen growth, providing a scientific basis for preventing and managing leaf blight in *C. lancifolius*.

**Methods:**

Pathogens were isolated from the interface of healthy and infected leaf tissues and identified through morphological and molecular biological methods. Amplification and sequencing of three genomic DNA regions—internal transcribed spacer region, translation elongation factor 1-α, and glyceraldehyde-3-phosphate dehydrogenase of ribosomal DNA—were performed, followed by the construction of a phylogenetic tree. The biological characteristics of pathogens under various temperature and pH conditions and different nitrogen and carbon sources were analyzed using the mycelial growth rate method. The antifungal effects of 13 chemical agents were evaluated using the poisoned medium method and mycelial growth rate method. Changes in physiological and biochemical indicators post-infection were also assessed. An antagonistic experiment was conducted to screen for biocontrol bacteria.

**Results:**

A total of 29 potential pathogenic strains were isolated from infected leaf tissues, with Koch’s Postulates confirming *Stemphylium lycopersici* as a key pathogen causing the disease. Growth analysis of *S. lycopersici* revealed optimal growth at 20°C and pH 6, with lactose or maltose serving as the most suitable carbon source and histidine as the preferred nitrogen source. Among the 13 synthetic fungicides tested, strain DHY4 exhibited the greatest sensitivity to 400  g/L flusilazole. Significant differences (*p*  < 0.05) were observed in superoxide dismutase, phenylalanine ammonia-lyase, peroxidase, polyphenol oxidase, catalase, and malondialdehyde levels between treated and control groups 3  days post-inoculation. The biocontrol strain DYHS2, identified as a strain of *Bacillus subtilis*, demonstrated an inhibition rate of 51.80% against *S. lycopersici* in dual-culture experiments and showed a relative inhibition rate of 78.82% in detached leaf assays.

**Discussion:**

These findings provide valuable insights into the newly identified causal agent of leaf blight in *C. lancifolius* and its biological characteristics, underscoring the potential of *B. subtilis* DYHS2 and synthetic fungicides such as flusilazole as effective disease management strategies.

## 1 Introduction

*Cyclocodon lancifolius*, a plant from the Campanulaceae family, is known for its therapeutic properties, particularly in its roots, and has been used to invigorate qi, replenish deficiencies, dispel stasis, and alleviate pain. It is primarily used to treat injuries, physical fatigue, and intestinal colic. Beyond its use as a Chinese medicinal herb, the fruit of *C. lancifolius* can be consumed as a food product (Ma M. et al., 2023), owing to its taste and nutritional value (Liu Y. et al., 2024). The fruit is rich in crude polysaccharides, crude cellulose, crude fats, and other components (Ma M. J. et al., 2023) known for their antioxidant and antibacterial pharmacological properties. This plant is a unique Chinese herb, combining medicinal, edible, and health-promoting characteristics, making it highly promising for future development with significant market potential. With the increase in market demand, the cultivation of *C. lancifolius* has expanded, but frequent disease outbreaks have become a major concern (Dang et al., 2023). In particular, leaf spot disease is a severe infection in which infected leaves exhibit spots of varying sizes and shapes, often appearing along the edges or surfaces of the leaves. These lesions gradually expand and spread, eventually covering the entire leaf, causing discoloration, yellowing, and in severe cases, complete withering and death of the plant. This substantially affects the economic returns in commercial production. To date, the issue of controlling leaf blight in *C. lancifolius* remains unaddressed in both domestic and international research.

Chemical agents are currently the primary means of controlling leaf blight, but their excessive use can result in ecological imbalances, pesticide residue exceedance, and soil acidification. In contrast, biological control utilizes the diversity of microorganisms to directly or indirectly promote plant growth while reducing both the quantity and virulence of pathogens, thereby mitigating their harmful effects on plants (Rupp et al., 2016). In recent years, microbial agents have gained increasing prominence in the agricultural sector for controlling plant diseases, becoming a key component of modern agricultural practices (Sharf et al., 2021). For instances, Li et al. (2022) found that *B. subtilis* HAAS01, an antagonistic against Fusarium wilt in sweet potatoes, stimulates the production of endogenous hormones and works in conjunction with the upregulation of defense enzymes and related gene expression to protect plants from disease. This approach not only promotes plant growth but also significantly enhances the plants’ disease resistance to diseases (Dutilloy et al., 2024).

The objective of this study was to isolate and purify the pathogens responsible for leaf blight in *C. lancifolius*. The pathogen was accurately identified through a combination of morphological and molecular characterization, along with pathogenicity testing. Its biological properties were studied, and suitable synthetic fungicides were screened. Additionally, preliminary studies on the mechanisms of infection were conducted to offer theoretical support for the physical control of plant diseases. Biocontrol bacteria with antagonistic effects against the leaf blight pathogen were also screened and evaluated for their inhibitory potential. This research provides a scientific basis for the biological control of leaf blight in *C. lancifolius*.

## 2 Materials and methods

### 2.1 Materials and instruments

The leaves were obtained from the plantation of Guizhou University of Traditional Chinese Medicine, located at 26.38304084°N, 106.62105820°E and an elevation of 1,180 m above sea level. During the peak of *C. lancifolius* leaf blight in July 2022, three samples were collected from each experimental field. One sample from each field was taken from infected plants in three different orientations, and these were used for fungal species isolation. The leaves were placed in sterile self-sealing bags, labeled with their collection location and date, and stored in a refrigerator at 4°C for later use. The culture media used in this study included potato dextrose agar (PDA), Luria–Bertani (LB) medium, and Czapek–Dox medium. All kits were purchased from Beijing Solarbio Science & Technology Co., Ltd. Tebuconazole (430 g/L), azoxystrobin (250 g/L), prochloraz (450 g/L), copper quinolate (33.5%), myclobutanil (40%), thiophanate-methyl (70%), fludioxonil (25 g/L), flusilazole (400 g/L), hymexazol (98%), carbendazim (50%), thiram (30%), pyraclostrobine (250 g/L), and difenoconazole (10%) were purchased from Xingnong Pharmaceutical (China) Co., Ltd.

### 2.2 Isolation and purification of the pathogen

The isolation and purification of the pathogen from infected samples were performed using the tissue and single-spore isolation method (Chen et al., 2022). The infected samples were first rinsed thoroughly under running water. In a sterile environment, the samples were disinfected by immersing in 75% ethanol for 10–30 s, followed by treatment with 0.1% mercuric chloride solution for 1–3 min. After rinsing three times with sterile water, any excess moisture was removed using sterile filter paper. Small squares (0.5 cm) were cut from the interface between infected and healthy tissue and inoculated onto PDA medium. Once fungal hyphae appeared, colonies from the edges were subcultured onto fresh PDA medium using a 5-mm punch and incubated at 28°C under dark conditions. This process was repeated to obtain pure cultures, which were then stored in cryovials containing 30% sterile glycerol at −80°C for future use.

### 2.3 Morphological observation and pathogenicity testing of the pathogen

Representative strains of the pathogen were isolated and purified and then cultured on PDA medium for 5 days. A 5-mm punch was used to remove agar plugs containing fungal mycelia, which were then placed on the microwounded surfaces of healthy *C. lancifolius* leaves and covered with cling film. Agar plugs without mycelia were used as controls, with three replicates used for each treatment. The leaves were incubated at 25°C under 85% humidity and 12-h light/dark cycles, and water was sprayed as needed. The development of disease symptoms was monitored and recorded daily. Once lesions appeared on the leaves, symptoms were observed and analyzed. The infected leaves were subjected to another round of isolation and purification using the tissue and single-spore method to compare the morphological characteristics of the pathogen (Zhong et al., 2023).

The representative strains were revitalized and cultured for 5 days. Using a punch, plugs were removed from the edges of the colonies and inoculated onto PCA medium. These cultures were incubated at 25°C for 4–7 days under dark conditions, during which the morphological characteristics of colonies were observed and recorded. Spores (*n* = 50) and mycelial structures were examined under an optical microscope (Nasehi et al., 2014).

### 2.4 Molecular biological identification of the pathogen

DHY4 was selected as the representative strain for DNA extraction. Three genomic DNA regions, including ribosomal DNA internal transcribed spacer (ITS) (White et al., 1990), translation elongation factor 1-α (TEF), and glyceraldehyde-3-phosphate dehydrogenase (GAPDH), were amplified and sequenced using the primers ITS1/ITS4, EF1/EF2, and gpd1/gpd2 (Supplementary Table S1). The polymerase chain reaction (PCR) amplification conditions were as follows: predenaturation at 95°C for 5 min; followed by 35 cycles of denaturation at 95°C for 30 s, annealing for 30 s at the primer-specific annealing temperature, and extension at 72°C for 1 min; and final extension at 72°C for 10 min. The PCR products were sent to Sangon Biotech (Shanghai) Co., Ltd. for sequencing. The resulting sequences were aligned against the NCBI database using BLAST (Luo et al., 2024), and phylogenetic trees were constructed using the maximum likelihood method via MEGA 11.0 software to determine the species of the strains. The sequences obtained from this study were submitted to the NCBI’s GenBank database^1^ to obtain the corresponding accession numbers.

### 2.5 Effect of physical factors on the growth of pathogen

Two different base media were used. First, the pathogen was cultured on PDA medium at varying temperatures (5°C, 10°C, 15°C, 20°C, 25°C, 28°C, and 35°C) and pH levels (4, 5, 6, 7, 8, 9, 10, 11, and 12). Each treatment was replicated three times. After 7 days of incubation, colony diameters were measured using the cross method. Second, Czapek–Dox medium was used as the base, with different carbon sources (glucose, fructose, and lactose) substituted for the original carbon source, resulting in a medium with varying carbon compositions. Additionally, beef extract, yeast extract, and urea were used as nitrogen sources to replace the original nitrogen source, resulting in a medium with different nitrogen compositions. Agar plugs of the pathogen (5 mm diameter) were inoculated into the center of PDA plates and incubated at 25°C for 7 days, with each treatment replicated three times. Colony growth was observed, and colony diameters were measured using the cross method (Li et al., 2024).

### 2.6 Selection of synthetic fungicides for pathogen control

The inhibitory effects of various agents (Weber and Hahn, 2019) on the pathogen were evaluated using the poisoned medium method and the mycelial growth rate method (Naegele et al., 2022). Thirteen chemical synthetic fungicides that showed an inhibition rate of over 75% were dissolved and diluted with sterile water to prepare stock solutions at a concentration of 1.0 × 10^4^ μg/mL. From each stock solution, 500 μL was added to 49.5 mL of PDA medium, resulting in drug-containing PDA plates with a uniform concentration of 10 μg/mL. PDA plates containing different dilutions of the agents were prepared. Agar plugs, with a diameter of 5 mm, containing mycelia were inoculated at the center of these medicated plates and incubated at 25°C for 7 days. The inhibitory effects of each treatment were observed and recorded. Each synthetic fungicide was tested at five concentrations, with three replicates per concentration, using PDA without any agents as the control. After incubation, the diameters of pathogen colonies were measured using the cross method, and the relative inhibition rate, median effective concentration (EC_50_), and toxicity regression equation were calculated (Tang et al., 2022).


$$\mathrm{Percent mycelial inhibition}\;\left(\%\right)=\left[\frac{\begin{array}{l} \mathrm{Control group}\;\\ \mathrm{colony diameter}-\\ \mathrm{Treatment group}\;\\ \mathrm{colony diameter} \end{array}}{\begin{array}{l} \mathrm{Control group}\;\\ \mathrm{colony diameter} \end{array}}-5\;\mathrm{mm}\right]\times 100\%$$

### 2.7 Changes in physiological and biochemical indices during the infection of *Cyclocodon lancifolius* leaves with the pathogen

#### 2.7.1 Sample preparation and collection

The leaf blight pathogen strains were purified, and healthy *C. lancifolius* plants were selected for inoculation using the needle-punching method as described in section 2.2. Leaf samples, approximately 1 × 1 cm in size, were collected from the junction between infected and healthy tissue at 0, 1, 2, 3, 5, 7, 9, and 12 days post-cultivation. These samples were rapidly frozen in liquid nitrogen and stored at −80°C until further use. Uninoculated healthy *C. lancifolius* leaves served as the controls.

#### 2.7.2 Measurement of defense enzyme activities and malondialdehyde levels in *Cyclocodon lancifolius* leaves

The activity levels of defense enzymes, including phenylalanine ammonia-lyase (PAL), peroxidase (POD), catalase (CAT), superoxide dismutase (SOD), and polyphenol oxidase (PPO), as well as the content of malondialdehyde (MDA) in the leaves following infection with the leaf blight pathogen were measured using the respective assay kits (Tang et al., 2022).

### 2.8 Isolation, screening, and identification of biocontrol bacteria

#### 2.8.1 Isolation and purification of biocontrol bacteria

Healthy and infected *C. lancifolius* leaves were cut into 2-cm pieces and placed in conical flasks. The leaf pieces were rinsed once with sterile water, soaked in 75% ethanol for 60 s, treated with 0.1% HgCl_2_ for 1–2 min, and then rinsed three times with sterile water. After drying on sterile filter paper, the pieces were inoculated onto PDA medium and incubated in the dark at 28°C. To inhibit fungal growth, ampicillin at a 1:1,000 dilution was added when pouring of the plates. Once bacterial colonies developed around the leaf pieces, individual colonies with distinct morphologies were selected and streak-purified on LB agar plates. The purified strains were then inoculated into LB and cultured with shaking for 24 h to obtain purified bacterial suspensions (Zheng et al., 2024).

#### 2.8.2 Screening of biocontrol bacteria

The dual-culture plate method was employed to screen for antagonistic bacteria. Agar plugs of *S. lycopersici*, 5 mm in diameter, were inoculated at the center of PDA plates. Bacterial isolates were symmetrically inoculated at four points equidistant points, 2 cm from the pathogen inoculum, using the cross-streaking method, with sterile water serving as a control. Each treatment was replicated three times and incubated at 28°C for 5 days, after which the radius of pathogen growth was observed and measured. Controls with an equivalent volume of sterile water were included, with each treatment repeated three times. The antibacterial activity of each strain was assessed, and the most active strain was selected for further experiments against leaf blight in *C. lancifolius*.

#### 2.8.3 Morphological and physiological-biochemical identification of biocontrol bacteria

The antagonistic strains identified in section 2.7.2 were streaked onto LB medium and incubated at 25°C for 3 days. Colony morphology was then observed, and the strains were preliminarily identified based on their morphological and physiological-biochemical characteristics.

#### 2.8.4 Molecular identification of biocontrol bacteria

The selected strains were inoculated into LB liquid medium and cultured at 30°C under shaking conditions at 180 rpm until they reached the logarithmic growth phase. Genomic DNA was then extracted, and PCR amplification of the 16S rDNA sequence was performed using the universal primers 27F (5′-AGAGTTTGATCCTGGCTCAG-3′) and 1492R (5′-TACGGYTACCTTGTTACGACTT-3′). The PCR program for amplifying the 16S rRNA gene fragment comprised the following steps: an initial denaturation at 94°C for 4 min and 30 s; followed by 30 cycles of denaturation at 94°C for 40 s, annealing at 53°C for 45 s, and extension at 72°C for 1 min and 20 s; and final extension at 72°C for 10 min. The PCR products were sequenced by Sangon Biotech (Shanghai) Co., Ltd. The resulting sequences were aligned against the NCBI database using BLAST and analyzed via MEGA11.0 software. A phylogenetic tree was constructed using the maximum likelihood method to determine the species of the strains.

#### 2.8.5 Evaluation of biocontrol strain efficacy

Healthy *C. lancifolius* detached leaves were wiped with 75% ethanol and placed in a Petri dish lined with moist filter paper. Six wounds were created on each leaf using a sterile needle. Five different treatments were applied at the wound sites: (i) Blank group: addition of distilled water followed by inoculation with a sterile PDA plug; (ii) Control group: addition of distilled water followed by inoculation with DHY4 strain agar plugs; (iii) Strain DHYS2 fermentation broth group: treatment with DHYS2 fermentation broth (the strain was cultured in LB broth at 180 rpm and 30°C for 18 h): preparation of a DHYS2 spore suspension (1 × 10^6^ spores/mL), which was allowed to stand for 15 min before inoculation with DHY4 strain agar plugs; (iv) The leaves were treated with a solution of fluosilazole and allowed to stand for a period of 15 min prior to the inoculation of agar blocks containing the DHY4 strain. Each treatment was applied to nine leaves, and the experiment was repeated three times. Cotton moistened with water was placed over the plugs, and the Petri dishes were sealed with cling film. The dishes were incubated at 25°C under partial light conditions. After 7 days, lesion diameters were measured, and the inhibition rate of mycelial growth was calculated.


$$\mathrm{Inhibition rate}\;\left(\%\right)=\left[\frac{\begin{array}{l} \mathrm{Control lesion diameter}-\\ \mathrm{treatment group lesion diameter} \end{array}}{\begin{array}{l} \mathrm{Control lesion diameter}-\\ \mathrm{Blank group diameter} \end{array}}\right]\times 100\%$$

### 2.9 Statistical analysis

The experimental data were analyzed using analysis of variance—Duncan’s descriptive variance chi-squared test, which was performed via SPSS IBM 26.0, to ensure data normality. Enzyme activity data were also analyzed using SPSS 26.0, and statistical significance was assessed at *p* < 0.05. Images were processed using Photoshop 2022.

## 3 Results

### 3.1 Identification of the pathogen causing leaf blight in *Cyclocodon lancifolius*

Leaf blight, a highly contagious infection, is commonly observed in the seedlings of *C. lancifolius*; however, it can also affect adult leaves. Seedlings have a notably higher mortality rate than adult leaves. The disease typically begins at the leaf tips but can also develop along the edges and in the middle of the leaves. Early symptoms include yellowish-brown to dark brown areas that exhibit watery, irregular lesions. These lesions spread irregularly, causing the entire leaf to become infected, wilt, and die (Supplementary Figure S1).

A total of 29 strains were isolated from *C. lancifolius* leaves affected by leaf blight. Based on morphological characteristics, these strains were initially categorized into four types. The first type included 24 strains, accounting for 82.76% of the total isolates. These 24 strains were further subdivided into four types based on morphology and isolation timing, represented by strains DHY4, DHY5, DHY6, and DHY7. The second type comprised three strains, accounting for 10.34% of the total isolates. These three strains were further subdivided into one type, represented by DHY11. The third type included two strains, accounting for 6.9% of the total isolates. These strains were further subdivided into one type, represented by DHY12 (Supplementary Figure S2).

**Figure 1:**
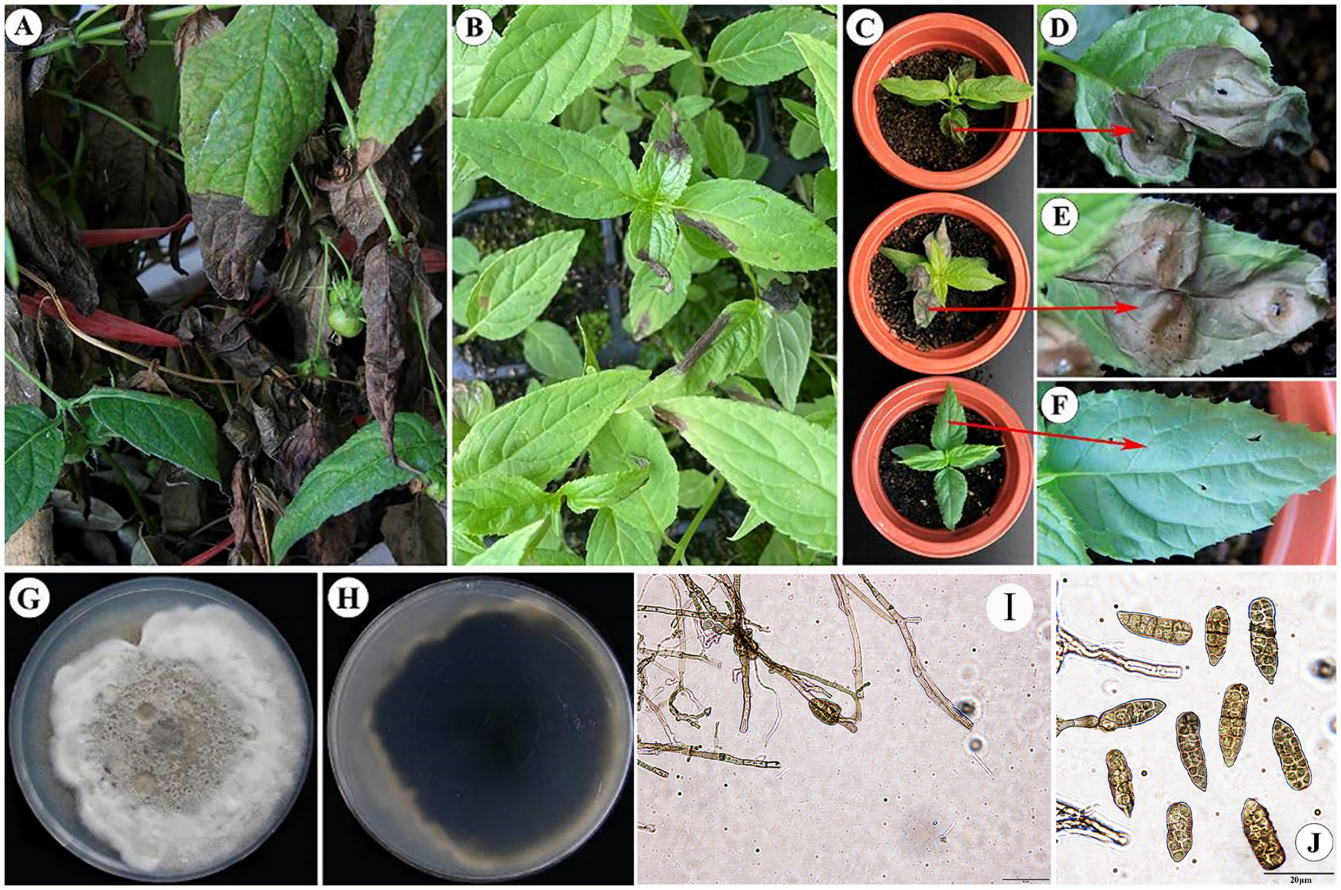
Symptoms and morphological characteristics of leaf blight in *C. lancifolius*.

The experiments demonstrated that only the first type of fungi caused leaf blight in sterile *C. lancifolius* seedlings. Fifteen days after inoculation, all inoculated leaves showed symptoms similar to those observed in the field, whereas the control leaves remained asymptomatic. Pathogens were re-isolated from the infected *C. lancifolius* leaves, and the isolates obtained post-isolation showed symptoms similar to the strains purified before inoculation. The pathogenicity tests confirmed that the strains DHY4, DHY5, DHY6, and DHY7 are pathogenic and responsible for leaf blight in *C. lancifolius* (Figures 1A–F).

The representative strain of the pathogen causing *C. lancifolius* leaf blight, DHY4, produced brownish-tan or white powdery mycelium on the culture medium, with brownish-tan honeycomb-like patterns in the center. The conidia were rod-shaped to conical and brown in color, with 1–7 transverse septa and 1–2 longitudinal septa. The spores measured 15.2630.14 × 7.1513.28 μm (*n* = 50), (Figures 1G–J). These morphological features were consistent with those described for *S. lycopersici*; thus, strain DHY4 was preliminarily identified as *S. lycopersici*.

**Figure 2:**
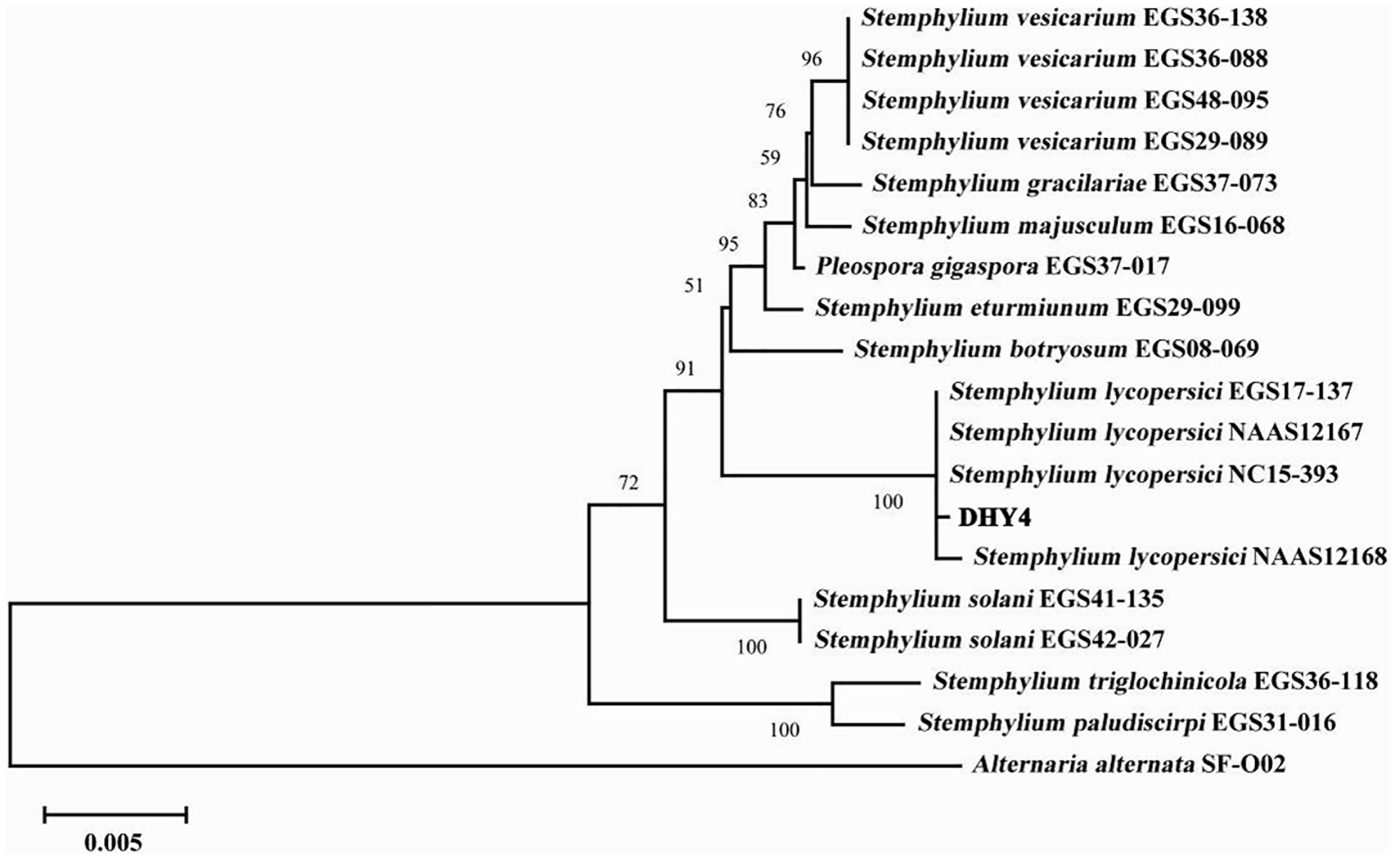
ML phylogenetic tree of strain DHY4. Phylogenetic analysis of concatenated sequences of the ITS region, TEF and GAPDH obtained from *S. lycopersici* DHY4 from this study and reference sequences of *Stemphylium spp*. specimens using the maximum likelihood method (1000 bootstrap iterations). *Alternaria alternata* SF-O02 was used as the outgroup. Bootstrap values are provided next to the respective branches.

Strain DHY4 was selected for DNA extraction. The primers ITS1/ITS4, EF1/EF2, and gpd1/gpd2 were used to amplify the ribosomal DNA ITS, TEF, and GAPDH, respectively, which were then sequenced. BLAST searches revealed that all sequenced regions showed 99–100% similarity with corresponding sequences of *S. lycopersici*. Using MEGA 11 software, a maximum likelihood phylogenetic tree was constructed based on the combined sequence data of ITS, TEF, and GAPDH regions using three sets of primers. Strain DHY4 clustered with the type species of *S. lycopersici* and was clearly separated from other species within the same genus. Based on both morphological characteristics and molecular biology features, the pathogenic strains DHY4, DHY5, DHY6, and DHY7 were identified as *S. lycopersici* (Figure 2; Supplementary Table S2).

### 3.2 Testing of biological characteristics of the pathogen

**Figure 3:**
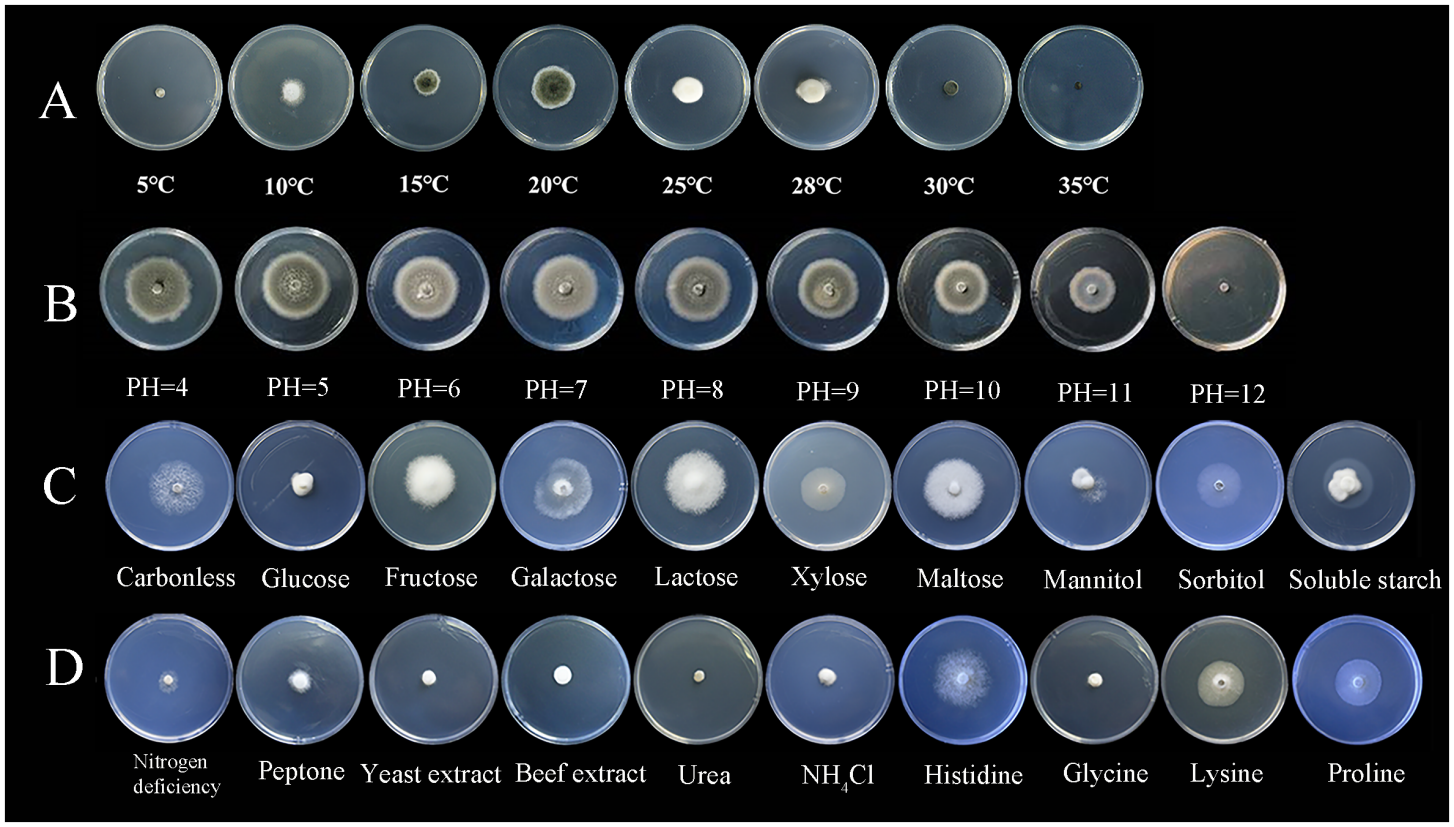
Growth of the pathogen under various conditions.

**Figure 4:**
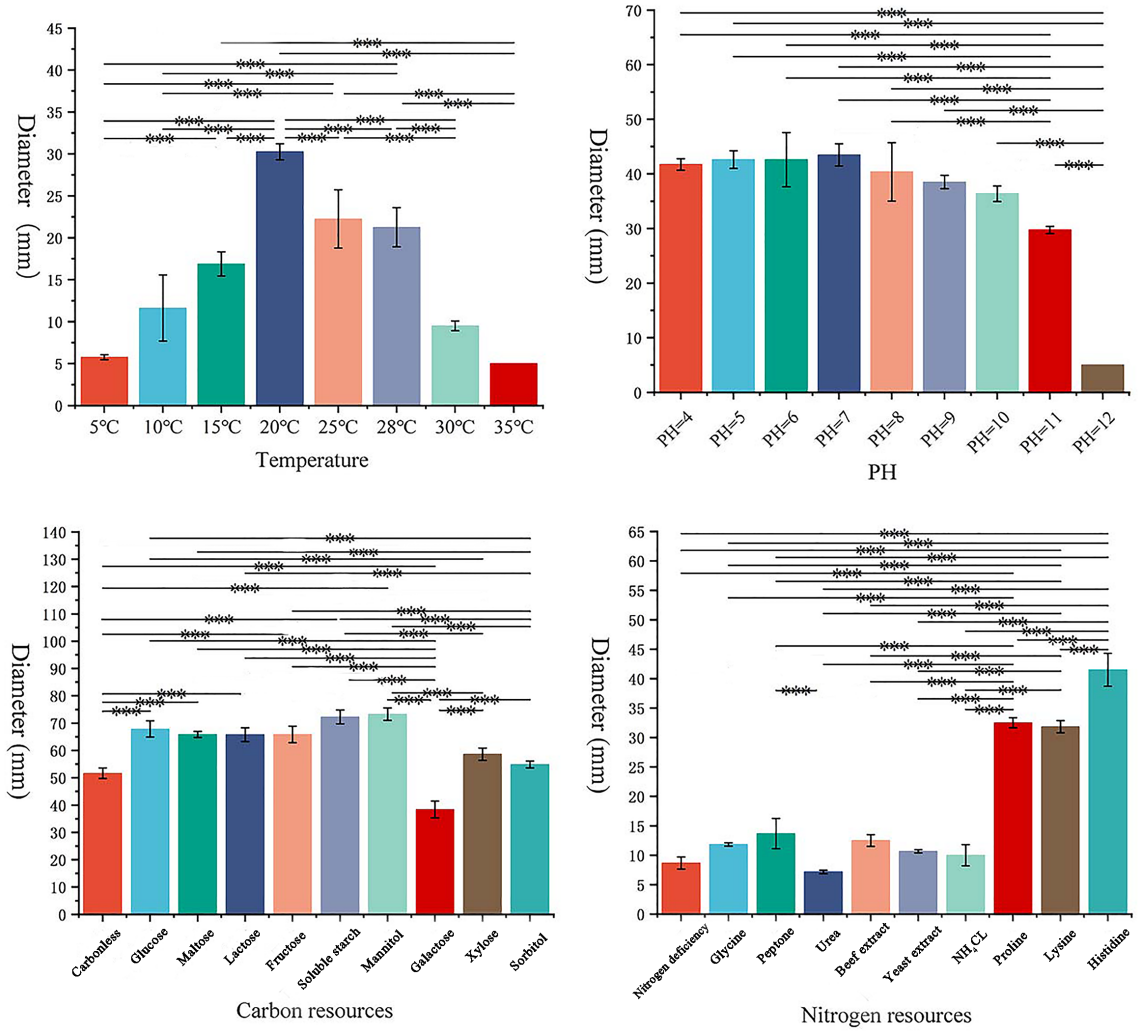
Effects of different conditions on the growth of pathogens.

*S. lycopersici* can grow at temperatures ranging from 5°C to 35°C, with optimal growth occurring at 25°C–28°C. Growth was significantly inhibited below 15°C. Among the eight temperature gradients tested, overall growth was slow, with the highest growth observed at 20°C, followed by 25°C, and reduced growth at other temperatures. The pathogen can thrive in pH conditions ranging from 4 to 12, with neutral pH around 6 being most conducive to growth. Significant differences in growth were noted across these nine pH gradients, with slightly acidic conditions favored, while growth was minimal at the highly alkaline pH of 12. The pathogen grew on all tested carbon sources, showing more robust mycelial growth compared to conditions without any carbon source. Significant differences were observed among these nine carbon sources, with lactose and maltose supporting the best growth, followed by galactose, while other carbon sources were less effective. Similarly, the pathogen was able to grow on all tested nitrogen sources, demonstrating more vigorous growth compared to conditions lacking any nitrogen source, which was clearly unsuitable. Significant differences were found among the nine nitrogen sources tested; those containing histidine were most suitable, followed by proline and lysine, with other sources being less effective (Figures 3, 4).

### 3.3 Selecting of fungicidal agents

**Figure 5:**
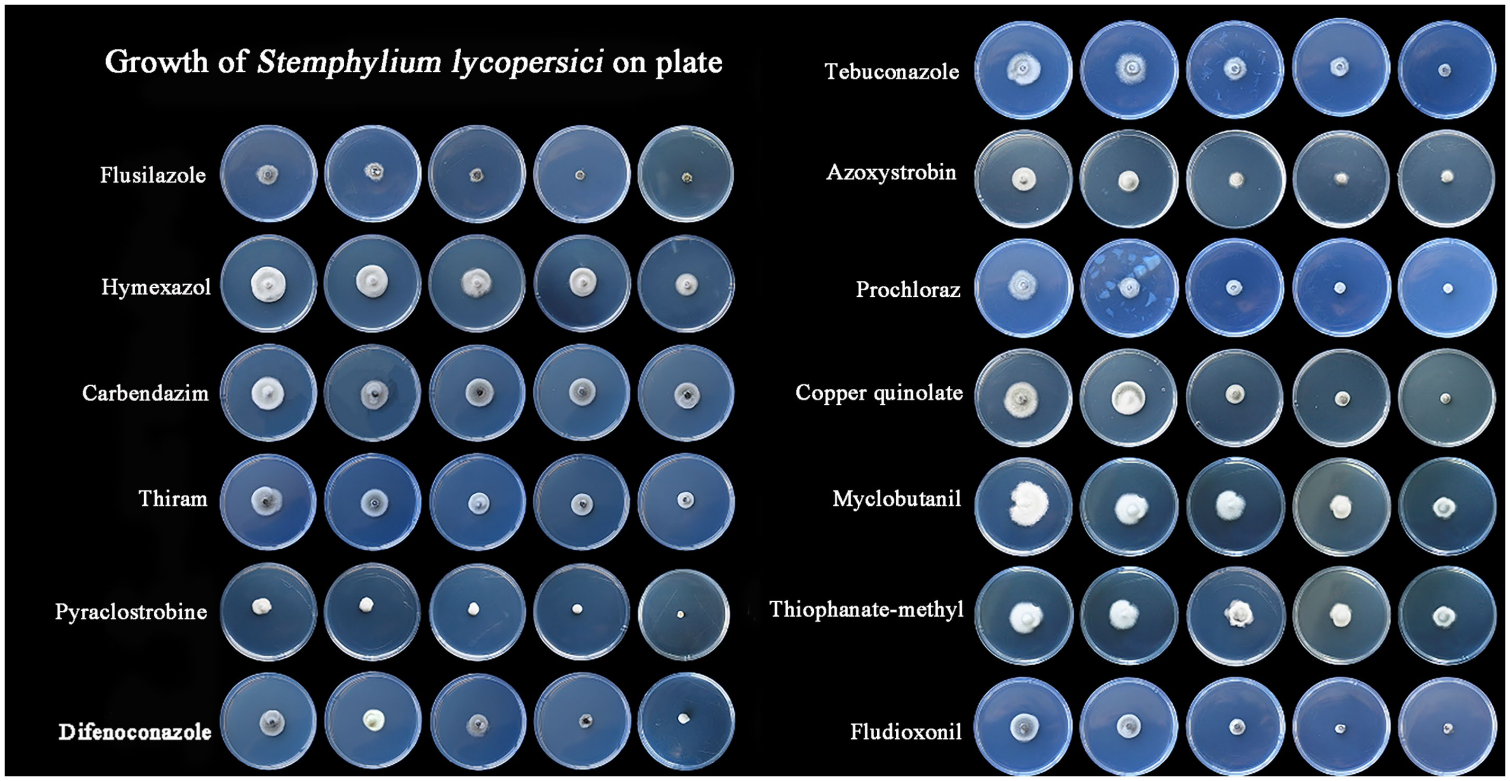
Mycelial growth of strain DHY4 in indoor toxicity test.

The most effective fungicide against the leaf blight pathogen DHY4 was 25 g/L fludioxonil, with an EC_50_ value of 1.49 μg/L. This was followed by 450 g/L prochloraz, 250 g/L pyraclostrobine, 40% myclobutanil, 430 g/L tebuconazole, 400 g/L flusilazole, 10% difenoconazole, and 250 g/L azoxystrobin, which also demonstrated strong inhibitory effects, with EC_50_ values of 2.39, 2.45, 3.18, 3.22, 3.62, 4.23, and 5.36 μg/L, respectively. Fungicides with moderate inhibitory capacity included 50% carbendazim, 33.5% copper quinolate, and 30% thiraw, with EC_50_ values of 79.75, 90.65, and 95.86 μg/L, respectively. The least effective fungicide was 70% thiophanate-methyl, with an EC_50_ value of 4055.39 μg/L, whereas 98% hymexazol was the least effective overall, with an EC_50_ value of 17502.03 μg/L. Toxicity testing revealed that the slopes of the toxicity regression equations for the 13 agents against strain DHY4 ranged from 0.40 to 50.1, with 400 g/L flusilazole showing the highest slope. This indicates that strain DHY4 is most sensitive to 400 g/L flusilazole among the tested fungicides (Figure 5; Table 1).

**Table 1:** Regression equations and EC_50_ information for the growth of strain DHY4 fungal mycelium against 13 fungicides.

Germicide	Toxicity regression equation	Correlation coefficient (*R*^2^)	EC_50_ (μg/L)	Activity ranking
Tebuconazole	*y* = 23.9*x* − 44.2	0.987	3.22	5
Azoxystrobin	*y* = 15.87*x* − 31.1	0.996	5.36	8
Prochloraz	*y* = 49.7*x* − 53.5	0.981	2.39	2
Copper quinolate	*y* = 22.4*x* − 46.9	0.977	90.65	10
Myclobutanil	*y* = 19.7*x* − 22.0	0.986	3.18	4
Thiophanate-methyl	*y* = 0.4*x* + 3.78	0.987	4055.39	12
Fludioxonil	*y* = 42.8*x* − 39.8	0.979	2.02	1
Flusilazole	*y* = 50.1*x* − 130	0.989	3.62	6
Hymexazol	*y* = 9.38*x* − 0.26	0.995	17502.03	13
Carbendazim	*y* = 13.6*x* − 27.4	0.986	79.75	9
Thiram	*y* = 17.3*x* − 35.2	0.984	95.86	11
Pyraclostrobine	*y* = 20.19*x* + 15.2	0.982	2.45	3
Difenoconazole	*y* = 31.4*x* − 74.8	0.989	4.23	7

### 3.4 Changes in physiological and biochemical indices during infection with the pathogen

**Figure 6:**
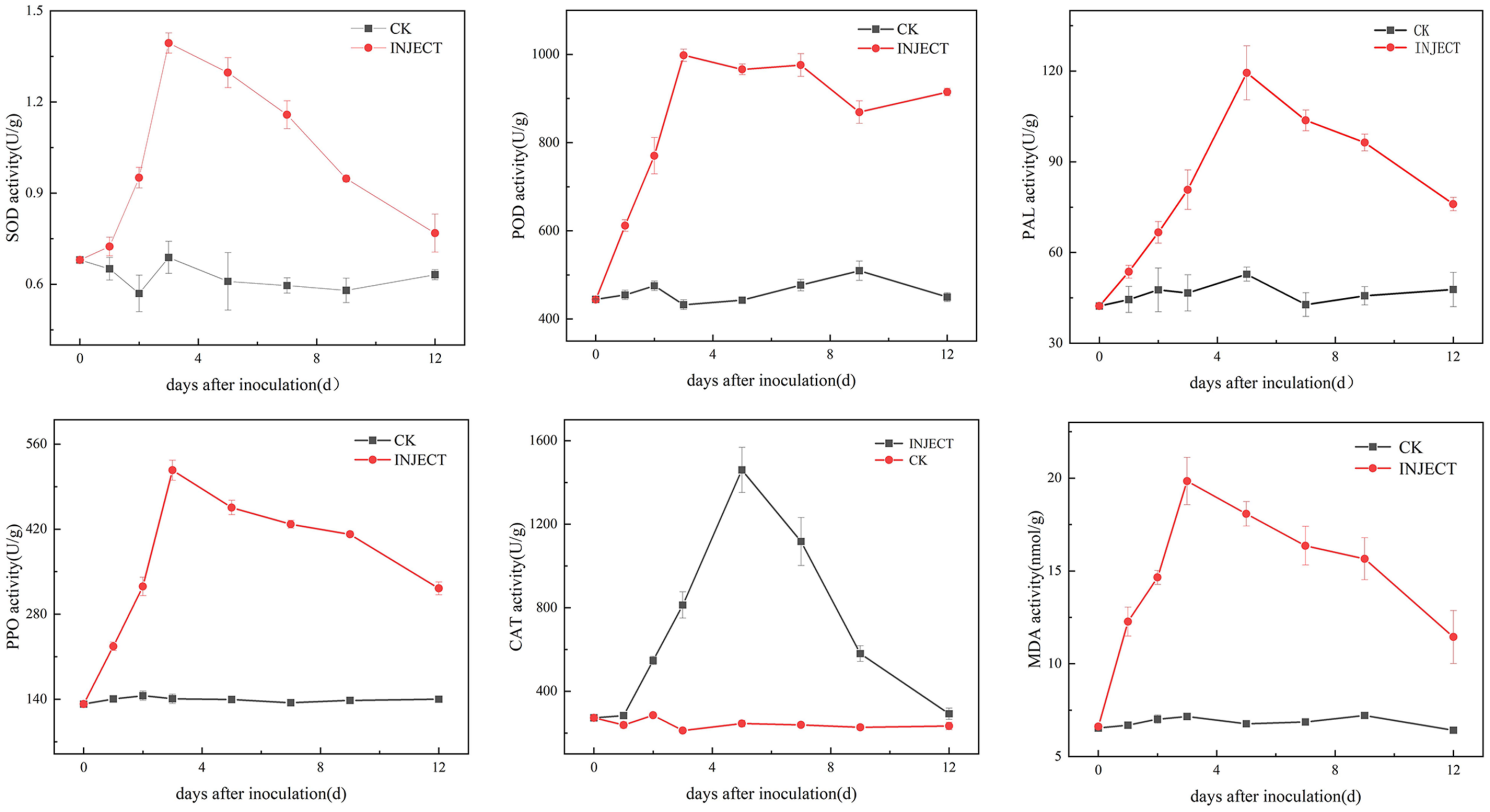
Changes in physiological and biochemical indicators during the infection process of strain DHY4 in *C. lancifolius*.

The activity levels of five defense enzymes (CAT, SOD, POD, PPO, and PAL) and the content of malondialdehyde (MDA) showed significant differences between the infected and control groups 3 days post-inoculation (Supplementary Figure S3). The results showed that, compared with the control group, the activity levels of SOD, POD, and PPO increased significantly on the third day after inoculation with the pathogen DHY4, reaching peak values of 1.394, 998.15, and 517.08 U/g, respectively. Subsequently, the activity levels of SOD and PPO began to decline, whereas the activity level of POD stabilized. Conversely, the activity levels of PAL and CAT continued to rise, significantly exceeding control levels. On the fifth day post-inoculation, the activity levels of PAL and CAT peaked at 119.41 and 1459.90 U/g, respectively, before gradually declining to levels similar to those of the control group. The MDA content increased throughout the inoculation period. On the third day post-inoculation, the MDA content in *C. lancifolius* leaves under leaf blight stress increased significantly to 19.84 nmol/g, which was 2.77 times higher than that in the control group. Throughout the disease progression, the MDA content in the infected group remained higher than in the control group (Figure 6).

### 3.5 Selection of biocontrol bacteria

**Figure 7:**
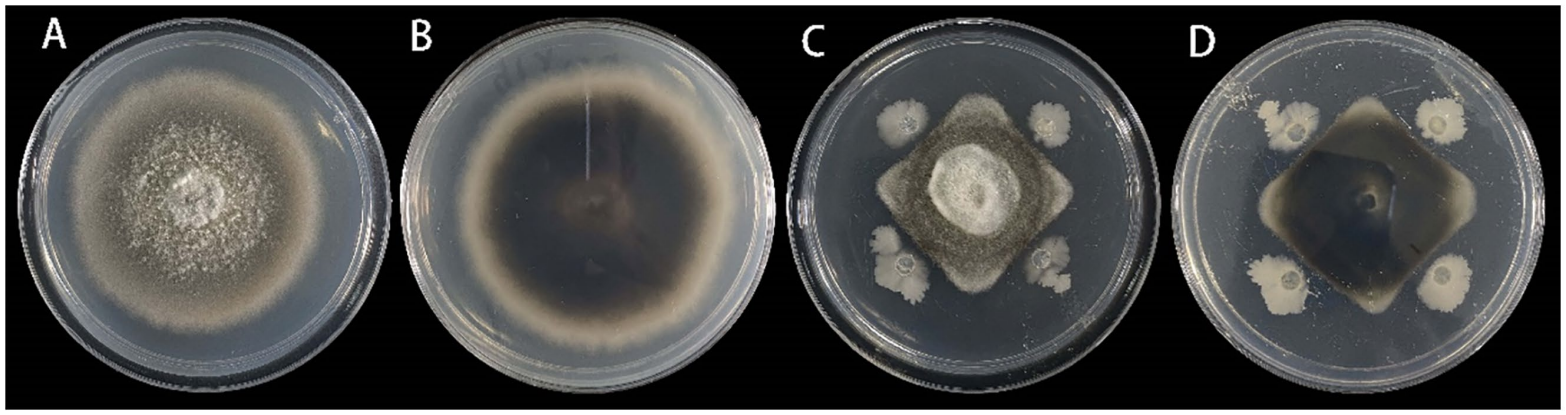
Inhibitory effect of strain DHYS2 on *S. lycopersici*.

From the leaf samples, 16 bacterial strains were isolated. After testing for antagonistic activity against *S. lycopersici*, four strains were preliminarily identified as having antagonistic effects. Among these, strain DHYS2, purified on LB medium, demonstrated the highest inhibitory effect against the pathogen (Figure 7), with an inhibition rate of 51.80% and an average inhibition zone of 0.43 cm.

**Figure 8:**
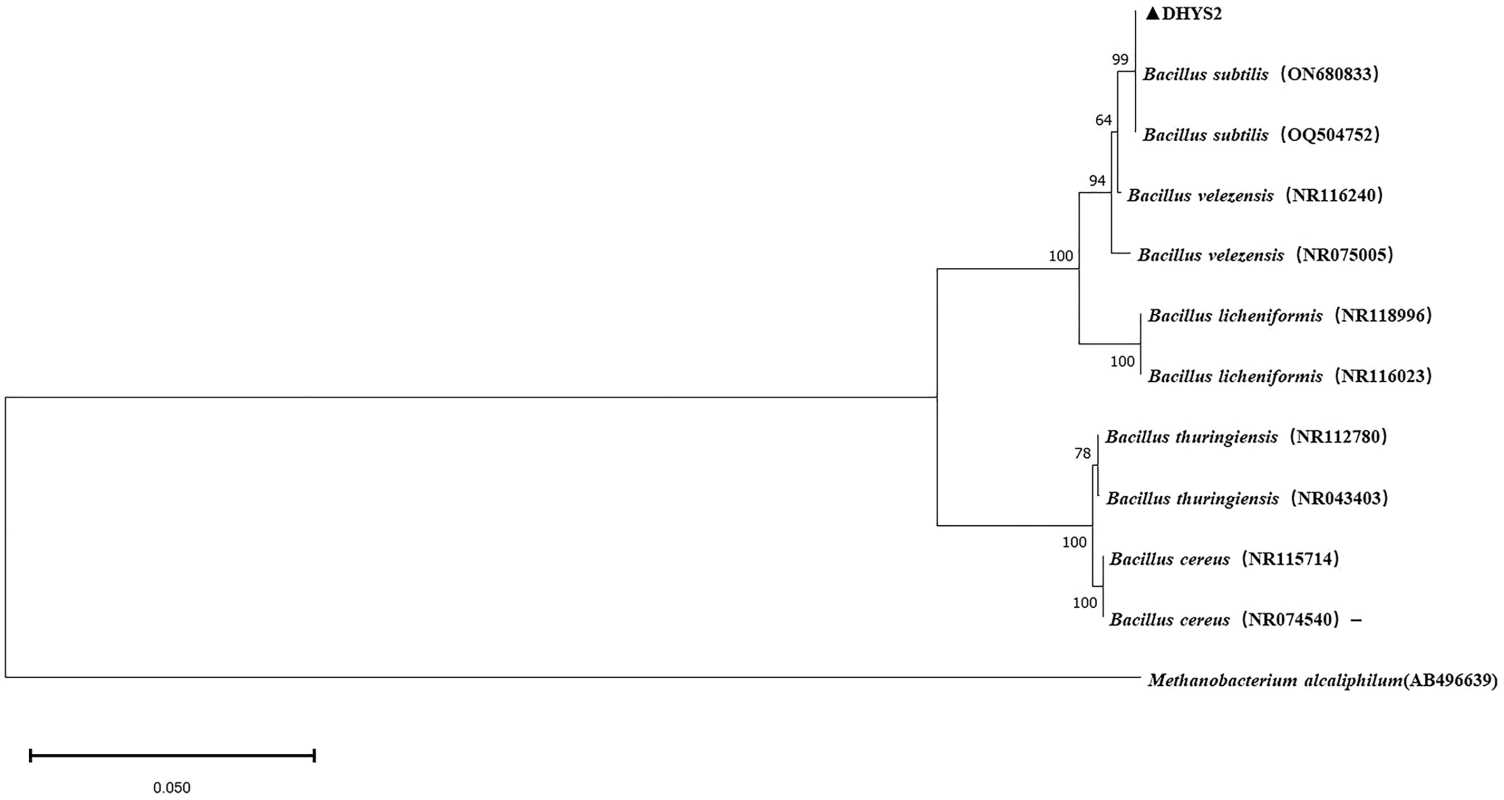
Phylogenetic tree of strain DHYS2 based on 16S rDNA sequence. Phylogenetic analysis of concatenated sequences from *B.subtilis* from this study and reference sequences of Bacillus spp. Specimens using the maximum likelihood method (1000 bootstrap iterations). *Methanobacterium alcaliphilum* AB496639 was used as the outgroup. Bootstrap values are provided next to the respective branches.

On LB medium, colonies of strain DHYS2 initially appeared round or nearly round, with a wet and glossy surface that was difficult to scrape and translucent in appearance. Over time, the colony surfaces became dry and wrinkled, gradually changing to an off-white color. Gram staining confirmed positive results (Supplementary Figure S4). Strain DHYS2 can grow under the following conditions: temperature, 20°C–41°C; pH, 5.7; and salt concentrations, 2–7%. The strain showed growth on D-xylose, L-arabinose, D-mannitol, and citrate and tested positive for Voges–Proskauer test, gelatin liquefaction, nitrate reduction, and starch hydrolysis. However, it tested negative for acetate utilization and anaerobic growth (Supplementary Table S2). Based on morphological characteristics, physiological and biochemical results, strain DHYS2 was preliminarily identified as similar to *Bacillus subtilis*. A phylogenetic tree was constructed using the 16S rDNA sequence of strain DHYS2, along with 12 other Bacillus species, showing that strain DHYS2 is closely related to *Bacillus subtilis*, with 100% similarity to *B. subtilis* (ON680833). Therefore, strain DHYS2 (NCBI upload number: PP868491) was confirmed as *Bacillus subtilis* (Figure 8).

**Figure 9:**
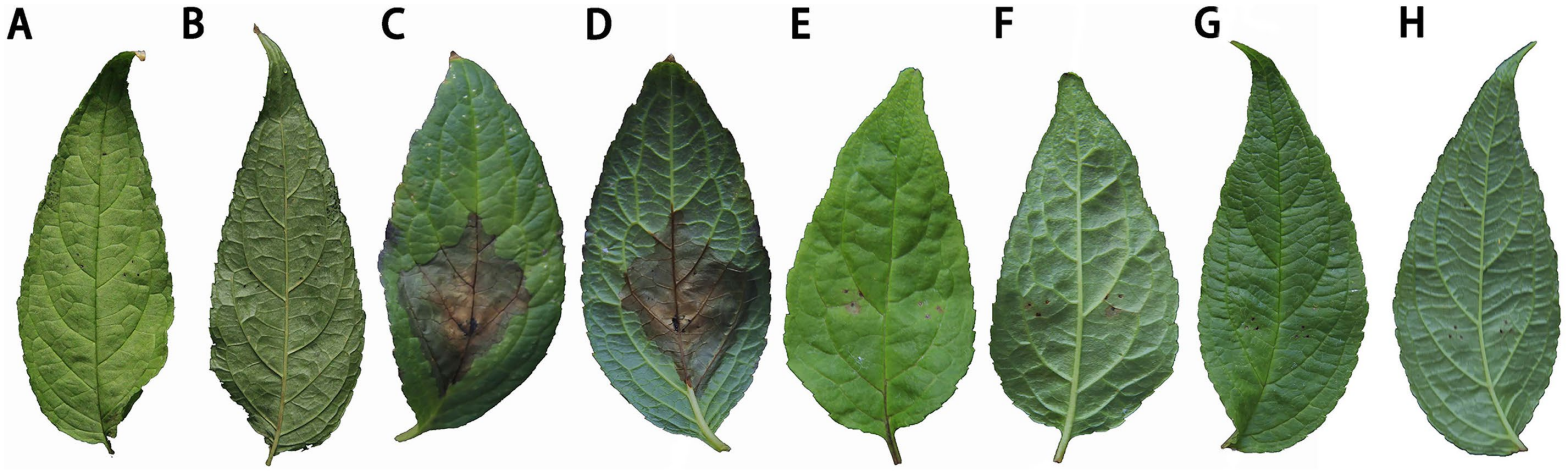
Comparison of treatment efficacy on detached leaves. **(A-B)**: blank group (spraying distilled water to inoculate sterile PDA block); **(C-D)**: control group (spraying distilled water to inoculate strain DHY4 bacterial cake); **(E-F)**: Strain DHYS2 fermentation broth group (spraying strain DHYS2 fermentation broth to inoculate strain DHY4 bacteria cake); **(G-H)**: Flusilazole group (spraying flusilazole to inoculate strain DHY4 bacteria cake).

The control effects on *C. lancifolius* leaves are summarized in Figure 9. Seven days post-treatment, significant differences were observed in the diameters of lesions between the treatment groups and control and blank groups. Lesions in the control group were significantly larger than those in the groups treated with DHYS2 fermentation broth and flusilazole, indicating that both DHYS2 fermentation broth and flusilazole exert significant inhibitory effects on the pathogen. The inhibition rates in the groups treated with DHYS2 fermentation broth and flusilazole were 78.82 and 88.82% (Table 2), respectively, with no significant differences observed between the two treatments. The control group displayed typical yellowish-brown, nearly circular lesions, whereas the blank group showed only a few brown spots at the wound sites.

**Table 2:** The antimicrobial effect of antagonistic bacteria on pathogens on leaf.

Treatment	The average lesion size/cm	Relative inhibition rate /%
Blank group	—	—
Control group	1.53 ± 0.196b	—
Fermentation broth group of strain DHYS2	0.324 ± 0.244a	78.82 ± 1.29a
Flusilazole group	0.171 ± 0.372a	88.82 ± 0.45a

Different lowercase letters after the same column indicate significant differences between different treatments (*p* < 0.05).

## 4 Discussion

Stemphylium is a globally distributed plant pathogen that inflicts damage on plant cells by producing and releasing toxic secondary metabolites, leading to disease. It can cause severe conditions such as leaf spot in various plants, including tomatoes, peppers, eggplants, lettuce, and persimmons (Torres et al., 2024). The disease typically presents as irregular, haloed, brownish spots on leaf tips and edges. In *C. lancifolius*, the disease manifested as a yellow-brown to dark brown, watery ring of lesions on the leaves, which is consistent with the symptoms previously reported for leaf blight (Chen et al., 2022). This study is the first report of *S. lycopersici* affecting *C. lancifolius* in China, further expanding the known host range of this pathogen.

The outbreak and spread of the pathogen are influenced by multiple factors, with temperature being a critical environmental factor affecting its growth, infection, and pathogenic processes (Liu C. et al., 2024). Different temperatures can impact the pathogen’s virulence. In addition to temperature, environmental factors such as carbon and nitrogen sources also play a role in pathogen infection. Currently, there is no report on the biological characteristics of *S. lycopersici*. Nevertheless, *S. lycopersici* shows similar growth patterns to other vegetative pathogenic fungi, exhibiting the greatest vigor under acidic conditions and at temperatures between 25°C and 28°C. Therefore, with temperatures rising to 25°C after the cold season, it is essential to prepare for potential disease outbreaks and enhance agricultural management. Although the pathogen thrives at elevated temperatures, this study found that *S. lycopersici* struggles to grow at the high temperature of 35°C, further confirming that leaf blight in *C. lancifolius* typically occurs during the seedling stage in May.

Presently, the primary method of preventing and controlling agricultural diseases is the use of chemical synthesis agents (Cowger et al., 2019). The advantages of these chemical methods include their rapid efficacy, ease of use, and low cost. However, the effectiveness of chemical agents in controlling *S. lycopersici* remains inconclusive. Consistent with other methods for managing pathogenic bacteria (Islam et al., 2021), various agents have demonstrated significant inhibitory effects on *S. lycopersici*. Among these, pyraclostrobin and azoxystrobin have been identified as the most effective, with flusilazole demonstrating the greatest sensitivity. Consequently, these agents may be considered preferred methods for controlling tomato creeping stem mold. Fluosilazole, in particular, exhibits the highest sensitivity, making it a prime candidate for disease prevention and control. In contrast, mancozeb showed less pronounced fungicidal effects and is not recommended for field use. This study did not evaluate mixtures of different fungicides. To develop scientifically and rationally sound spraying programs, further research is needed to determine the optimal mixing ratios of effective fungicides for achieving the best inhibition results (Hou et al., 2024). Additionally, since this study was conducted indoors, external environmental conditions, such as temperature, rainfall, and humidity, may affect fungicide efficacy differently from indoor conditions. Therefore, further field efficacy tests are required to develop more reliable treatment plans.

Plants respond to pathogen invasion by secreting defense enzymes (Philip et al., 2024) such as SOD, PAL, PPO, POD, CAT as well as secondary metabolites such as phenolic compounds (Ponsankar et al., 2023). MDA, a byproduct of membrane lipid peroxidation, serves as a key indicator of leaf tissue antioxidant capacity and the extent of plant aging. High MDA contents reflect strong lipid peroxidation and rapid membrane damage. The activity levels of the five defense enzymes (SOD, PAL, POD, PPO, and CAT) and the content of MDA showed significant differences between the infected and control groups 3 days post-inoculation, with most parameters rapidly increasing to peak levels. Under healthy conditions, reactive oxygen species such as O2^−^, H_2_O_2_, and •OH are maintained at low stable levels because of plant metabolic activities. However, pathogen infection disrupts this cellular homeostasis, leading to an increase in the levels of reactive oxygen species. By day 9, the accumulation of reactive oxygen species that are not effectively cleared diminishes the plant’s disease resistance (Hyder et al., 2024). Pathogens further damage the cells, resulting in visible brown lesions on leaf surfaces. Although *C. lancifolius* activates internal defense enzymes to combat the disease, its capacity to resist infection is limited. Thus, timely intervention upon noticing disease signs is crucial for achieving better prevention and control outcomes.

For plant disease control, traditional chemical methods (Montenegro et al., 2018) can lead to environmental pollution and pesticide residues. Plants harbor various microbes (Huang et al., 2024), many of which are beneficial for plant growth, development, and resistance to disease and stress (Hardoim et al., 2015). Bacteria from the genus Bacillus are among the most extensively studied beneficial microbes and play a critical role in promoting plant growth and enhancing plant resistance to diseases and stress (Rekha et al., 2018; Botlagunta and Babu, 2024). These bacteria are widely used as biocontrol agents (Noh et al., 2024; Prasad et al., 2024). In this study, strain DHYS2 was isolated from *C. lancifolius* leaves and preliminarily identified as *B. subtilis* through morphological observation, physiological and biochemical characteristics, and molecular identification. It exhibited strong inhibitory effects against the pathogen causing leaf blight in *C. lancifolius*, demonstrating a broad range of antibacterial activity. The use of this strain does not contribute to environmental pollution or pesticide residues, thus ensuring the quality and safety of the herbal materials (Elshaghabee et al., 2017). Additionally, it promotes plant growth and shows significant biocontrol potential, making it suitable for widespread application in the biological control of leaf blight in *C. lancifolius*.

## 5 Conclusion

In conclusion, the experiment confirmed the infection of *S. lycopersici* on *C. lancifolius* leaves and established a theoretical basis for the physical prevention and control of this disease. Although *B. subtilis* is a vital beneficial biocontrol bacterium, the mechanism by which it inhibits *S. lycopersici* growth needs further investigation to explore potential disease management strategies.

## Data Availability

The datasets presented in this study can be found in online repositories. The names of the repository/repositories and accession number(s) can be found in the article/Supplementary material.

## Footnotes

^1^ https://submit.ncbi.nlm.nih.gov/
