# Supplementary material for: Preliminary study on Cyclocodon lancifolius leaf blight and screening of Bacillus subtilis as a biocontrol agent
Source: Front Microbiol. 2024 Oct 15;15:1459868. doi: 10.3389/fmicb.2024.1459868 (PMC11519681; doi:10.3389/fmicb.2024.1459868)
Supplement: Supplementary file 1 [file Data_Sheet_1.docx]

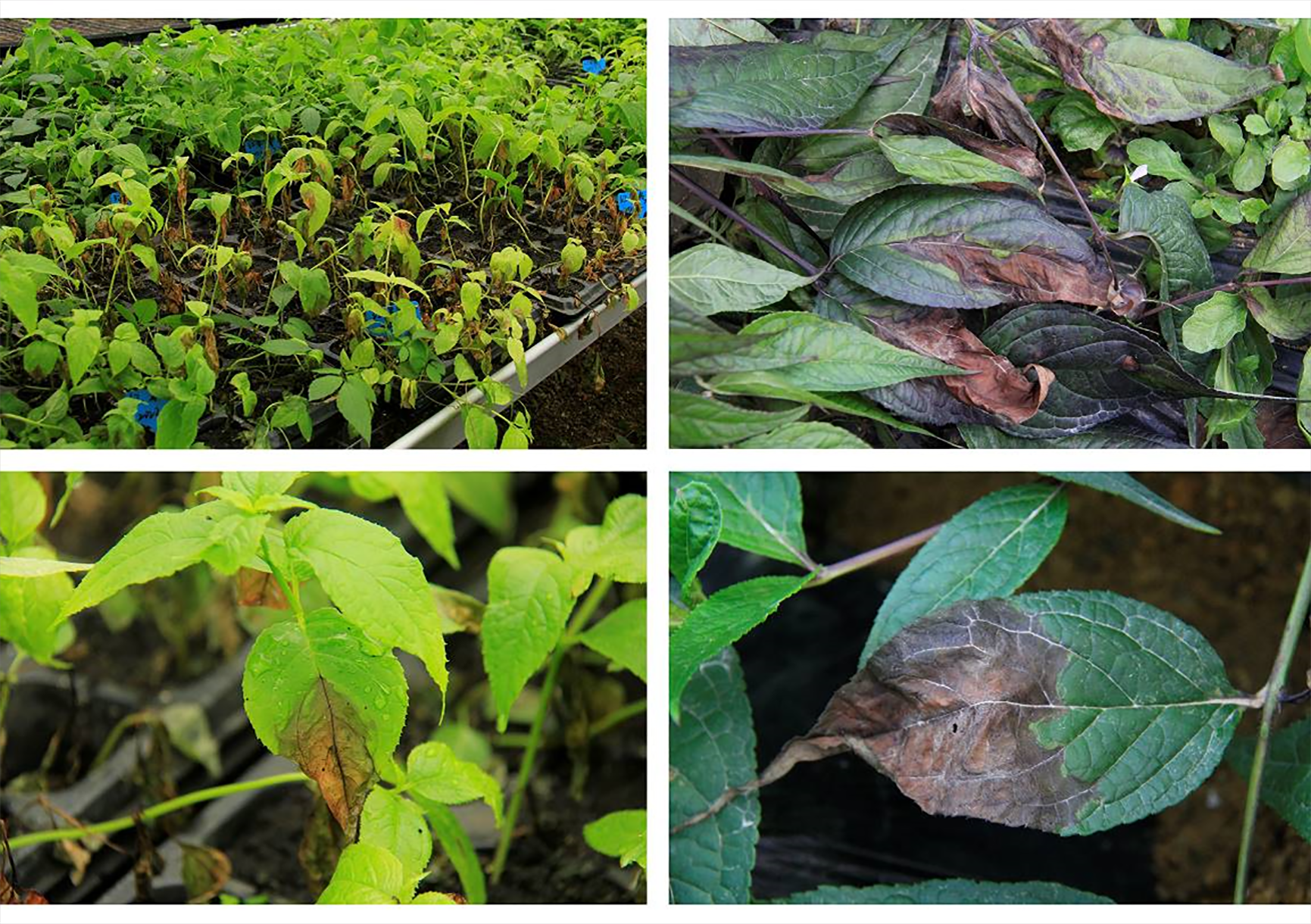


Fig S1. Symptoms of Leaf Blight in *C. lancifolius*


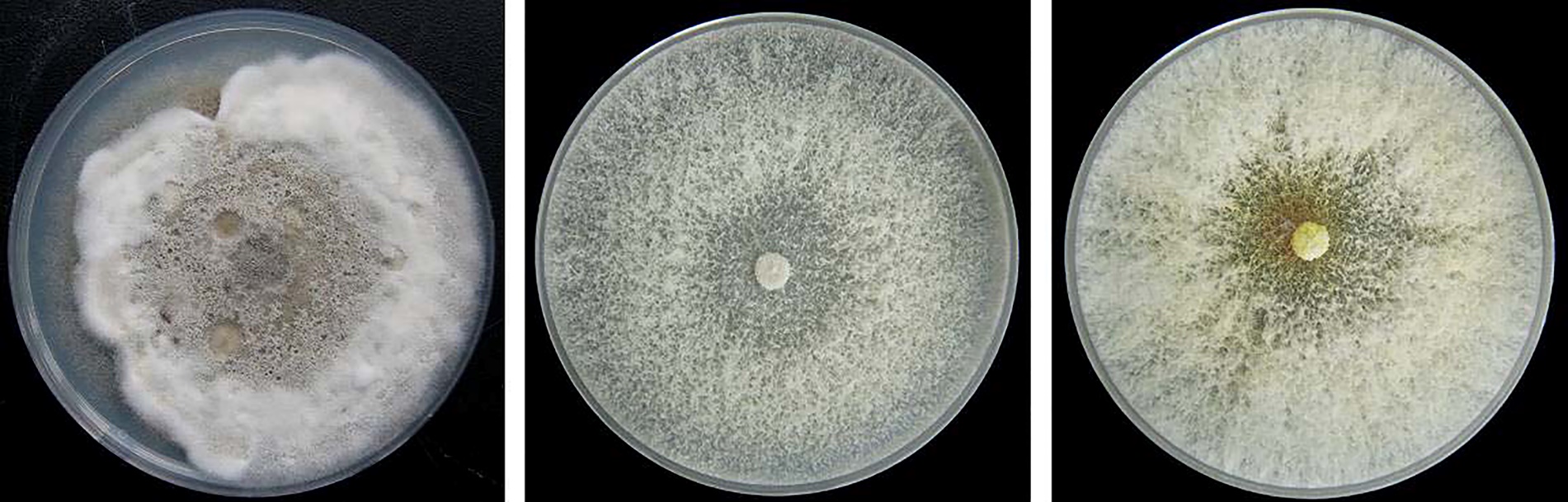


The first kind of fungi The second kind of fungi The third group of fungi

Fig S2. Morphologies of Three Types of Fungi Isolated from Leaf Blight-Affected *C. lancifolius* Leaves


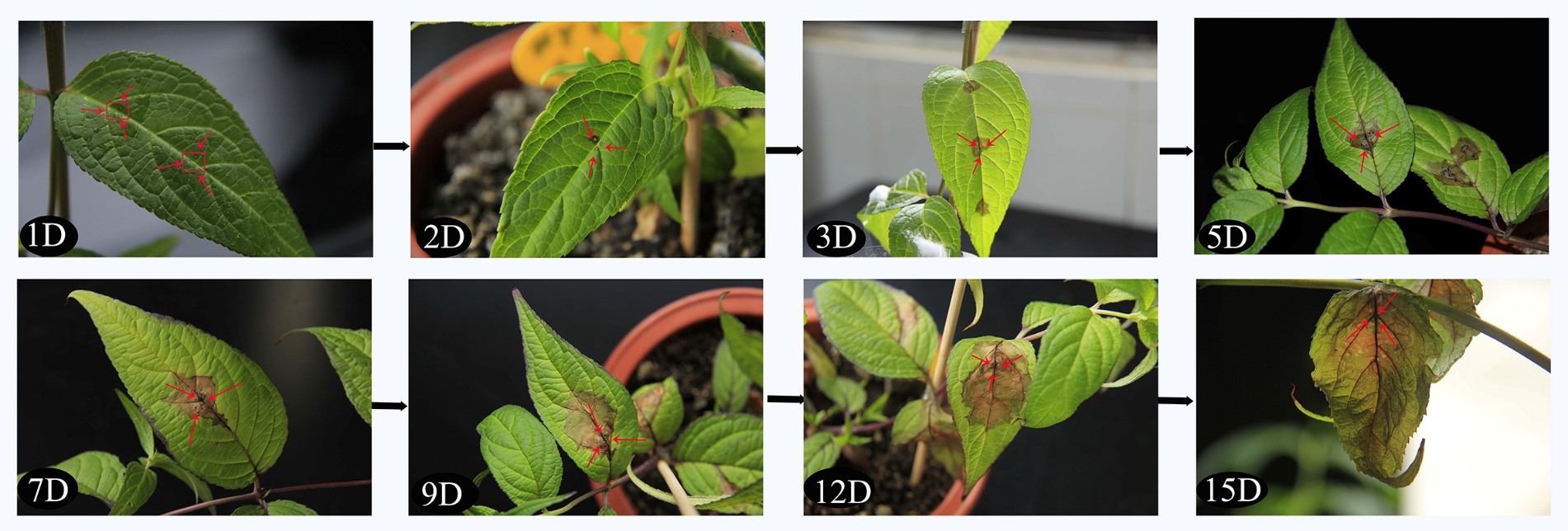


Fig S3: Process of Strain DHY4 Infecting *C. lancifolius* Leaves


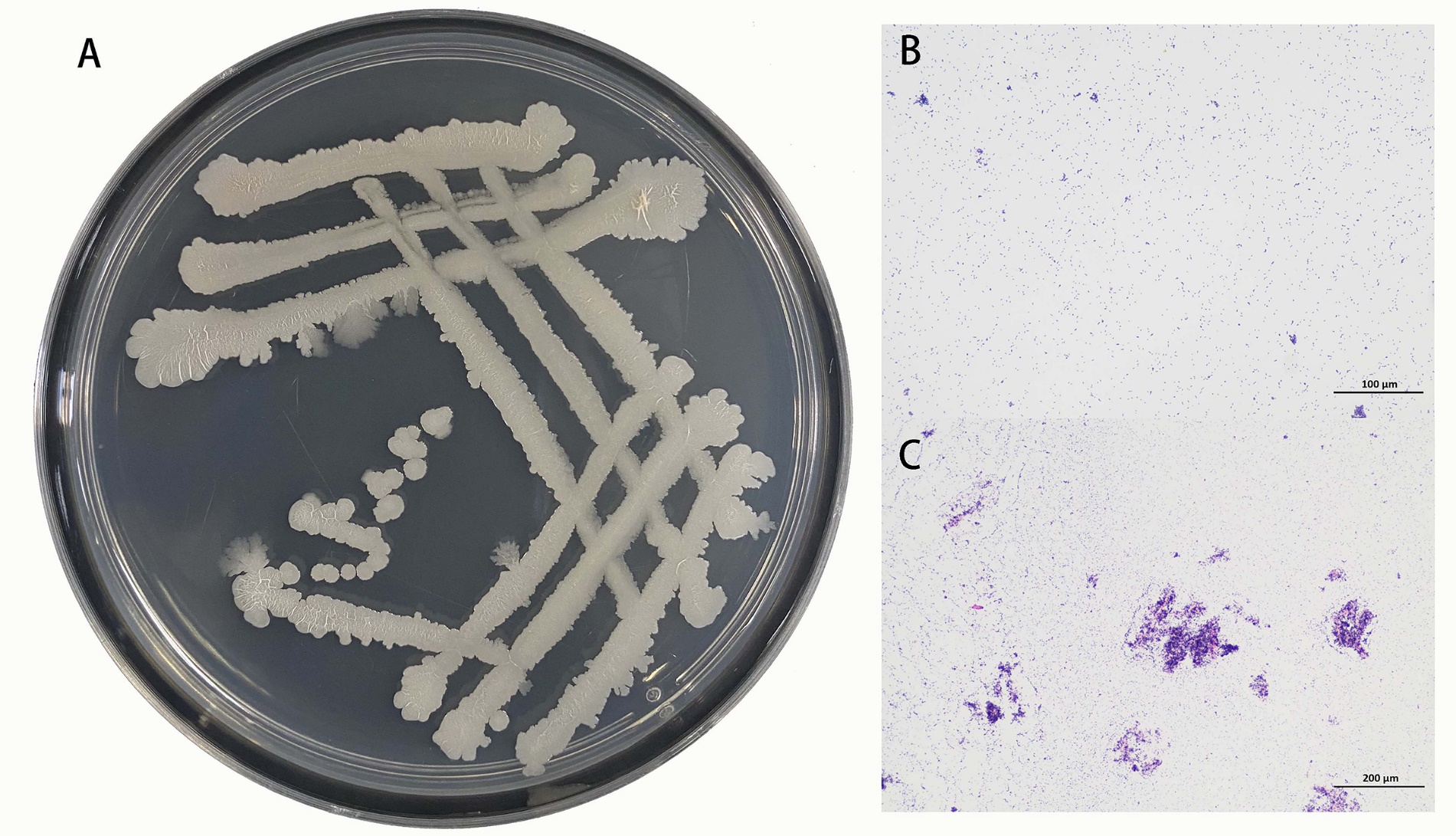


Fig S4: Colony Morphology of DHYS2 on LB Medium (A) and Gram Staining (B-C)

Table S1: DNA amplification primer sequence

| Gene | Primer | Primer sequences（5’-3’） | Annealing temperature /℃ |
| --- | --- | --- | --- |
| ITS | ITS1 | TCCGTAGGTGAACCTGCGG | 56 |
|  | ITS4 | TCCTCCGCTTATTGATATGC |  |
| GAPDH | gpd1 | CAACGGCTTCGGTCGCATTG | 56 |
|  | gpd2 | GCCAAGCAGTTGGTTGTGC |  |
|  | Alt-rev | ACGAGGGTGAYGTAGGCGTC |  |
| EF-α | EF1 | TGCGCTATTCTCATCATTGC | 53 |
|  | EF2 | AGAGGAGGGTAGTCAGTG |  |
|  | RPB1-R8 | CAATGAGACCTTCTCGACCAGC |  |

Table S2: Physiological and Biochemical Characteristics of DHYS2

| Identification project | Result | Identification project | Result |
| --- | --- | --- | --- |
| Anaerobic growth | - | 2%NaCl | + |
| Utilization of citrate | + | 5%NaCl | + |
| Gelatin liquefaction | + | 7%NaCl | + |
| Utilization of malonic acid | - | 10%NaCl | - |
| Voges-Proskauer (V-P) test | + | 4 ℃ | - |
| Starch hydrolysis | + | 10 ℃ | - |
| Nitrate reduction | + | 30 ℃ | + |
| D-xylose | + | 37 ℃ | + |
| L-arabinose | + | 50 ℃ | - |
| D-mannitol | + | pH 5. 7 | + |
|  |  | Chloramphenicol sensitivity | + |

Note : ' + ' indicates positive, the second column indicates color reaction, the fourth column indicates that the bacteria can grow, ' - ' indicates negative, the second column indicates no color reaction, and the fourth column indicates that the bacteria do not grow.
